# Design, Analysis and Experimental Investigations of a Double-Arm Based Micro-Gripper for Thin and Flexible Metal Wires Manipulation

**DOI:** 10.3390/mi13060925

**Published:** 2022-06-10

**Authors:** Yuezong Wang, Jiqiang Chen, Daoduo Qu

**Affiliations:** Faculty of Materials and Manufacturing, Beijing University of Technology, Beijing 100124, China; yaozongw@bjut.edu.cn (Y.W.); qudd@emails.bjut.edu.cn (D.Q.)

**Keywords:** micro-gripper, thin and flexible metal wires, bulk silicon technology, envelope traction

## Abstract

A robotic system for the automatic wire pulling of coreless motor winding is designed, including the design of an opening-closing control system and a micro-gripper’s tip structure with a double-armed elastic-beam structure for the support part and an enveloping clamping structure for the tip part. The micro-gripper captures the electrode wire from the root, encircles the wire after the envelope region is closed, and the thin and flexible electrode wire is pulled to the top of the electrode pad by the movement of the micro-gripper and released. The mechanical index of the micro-gripper is simulated to obtain the optimal structural parameters. The experimental results show that the electrode wire’s maximum bearing force is about 0.3 N. Under this reaction force, the deformation of the tip-envelope region of the micro-gripper is about 27.5 μm, which is sufficient for electrode wire pulling micro-manipulation. By comparison with the steel micro-gripper, the silicon micro-gripper has more advantages in shape integrity, machinability and mechanical properties.

## 1. Introduction

The coreless motor winding is an emerging product in the field of the motor. The coreless motor has the advantages of good control characteristics, fast response speed, high precision, small size, lightweight, and so on [1]. It is widely used in aerospace, military, medical equipment, intelligent robot and other high-tech fields [2,3,4]. In the manufacturing process of coreless motor, there have been a variety of related automation technologies, among which the wire pulling process of the coreless motor winding is a very important link before automatic welding. At present, the wire pulling manipulation of the coreless motor’s winding mainly relies on manual manipulation under the microscope. Because the electrode wire of winding is small in size and only tens of microns in diameter, the material of electrode wire is soft and the bearing force is small, the manual manipulation is difficult, and due to the physiological limit of workers and the factors of manipulation proficiency. As a result, the efficiency and consistency of manual wire pulling are defective. Therefore, it is of great significance to develop an automatic wire pulling robot system that can automatically capture the electrode wire and pull it to the pad position.

The existing methods of manipulation of thin and flexible wire mainly include the wind blowing method, direct clamping method, and so on, but the wind blowing method often has various problems in the actual manipulation process, and most of them are manipulated in the way of micro clamping. Micro clamping technology is different from ordinary clamping, handling and other manipulations. Its manipulation objects are usually small objects with sizes ranging from a few microns to a few hundred microns [5]. It has a good application prospect in micro-processing, micro-assembly, optics, medicine, biology and other fields [6]. Therefore, the research of micro clamping technology is an important solution to realize automatic manipulation. Xi et al. [7,8] studied the micromanipulation of linear metal wire by air blowing method. By placing the micromanipulator at a certain angle, the micromanipulator can continuously output airflow and continuously blow the wire to make the wire lie on the top of the pad. Wang et al. [9] designed an electromagnetically driven micro-gripper, which can be used to control the wire of diameter of 50–70 μm under the guidance of a Stereo Light Microscope (SLM) vision system clamping pulling manipulation. Wang et al. [10] designed a pneumatic micro-gripper, which uses the cylinder as the driving force element to realize the clamping and release manipulation. Wang et al. [11] designed a kind of motor-driven micro-gripper. The micro-gripper can be separately processed by precision machining technology and fixed into the system, which can realize the action of clamping and releasing. Alissa et al. [12] designed an electric micro-gripper embedded with a metal film heater based on SU-8, with a clamping stroke of 70–90 μm, which can be used to grip and place microsphere objects up to 150 μm in diameter. Uran et al. [13] proposed the use of capillary forces to reliably grasp and release tiny objects, which can be used to grasp glass beads between 5 and 60 μm in diameter or irregularly shaped dust particles of similar size with an accuracy of up to 0.5 μm. Zhang et al. [14] designed a V-shaped electrothermal driven five bar flexible micro-gripper for grasping, moving and releasing zebrafish embryos. Cauchi et al. [15] designed a horizontal electrothermal MEMS Micro-gripper with a maximum opening displacement of 9 μm. It is used to operate human red blood cells. Wang et al. [16] designed a piezoelectric driving micro-gripper, adopting a piezoelectric ceramic as the actuator, to achieve clamping operations on optical fibers with a diameter of 230 μm. The performance of micromanipulation is closely related to its driving mode. According to its driving mode, it is mainly divided into piezoelectric driving [17,18,19], electromagnetic driving [9], electrostatic driving [20], shape memory alloy [21,22], electrothermal driving [23], motor driving [24], etc. However, the cooling and heating time is needed in the manipulation process of electrothermal driven micro-gripper, and the response speed is slow; electromagnetic drive is difficult to reduce its scale, which is greatly affected by the external magnetic field, and due to the required components, it may lead to slow manipulation speed and reduce the system accuracy [25]; although piezoelectric drive has the advantages of high precision, light structure and fast response time [26], its opening-closing displacement is generally small, which is suitable for smaller objects and has the problem of hysteresis; the clamping force and opening-closing displacement of electrostatic driving are small; vacuum adsorption has higher requirements on the surface of objects; although the wind blowing method can effectively reduce the mechanical damage to the wires, the airflow is easy to interfere with the rest of the wires, resulting in operation failure. In addition, due to the small size and soft texture of the electrode wire, it is easy to damage the wire by using the ordinary straight arm micro-gripper, and the wire is flexible and does not show a regular line shape. The position of the suspension end is more random, and the clamping position has a greater impact on the manipulation. Because the shape of the wire is a curve with small deformation, it requires a large opening-closing displacement, and in order to achieve high quality and high efficiency, it requires fast manipulation speed, high frequency and high precision. These existing methods are not ideal for the pulling manipulation of thin and flexible wire, so the pulling problem of thin and flexible wire needs further optimization and in-depth study.

Before the automatic welding of the electrode wire of coreless motor winding, it is necessary to clamp and pull the electrode wire. To solve this problem, this paper designs a two-finger micro-gripper system for the manipulation of thin and flexible wire. In this system, the micro clamping method is used to pull electrode wire, so that the wire lies on the top of the pad to meet the requirements of automatic welding. In this paper, silicon materials are adopted to make a micro-gripper whose spatial motion and opening-closing motion are realized through combination with the lead screw drive in motor-driven mode. The micro-gripper system designed in this paper is very suitable for the clamping and pulling manipulation of the winding electrode wires of the coreless motor. The advantages are as follows: (1) The micromanipulator is etched with a silicon material, which has more advantages in shape integrity, machining accuracy and mechanical properties than precision machining. (2) Envelope-type design is adopted in the tip region. By capturing the root of the electrode wire, the micro-gripper moves to encircle the wire, and then the electrode wire is clamped and pulled to the upper part of the target pad by the movement of the micro-gripper and then released. The electrode wire is accurately captured without damage, which improves the accuracy and stability of traction to a certain extent. (3) The combination of motor driving and lead screw driving can achieve the characteristics of high precision, high response speed and large displacement. Due to the operation of the system, the pulling of the coreless rotor winding electrode wire improves both the quality and efficiency of the winding. More than that, the welding process is ensured to be automatic which, to some extent, increases the processing efficiency of products.

The remainder of this article is organized as follows: In Section 2, the materials and methods are designed; in Section 3, the experiments are carried out; in Section 4, the discussion is carried out. In Section 5, the conclusions are derived.

## 2. Materials and Methods

### 2.1. Function Analysis of Micro-Gripper

**Figure 1:**
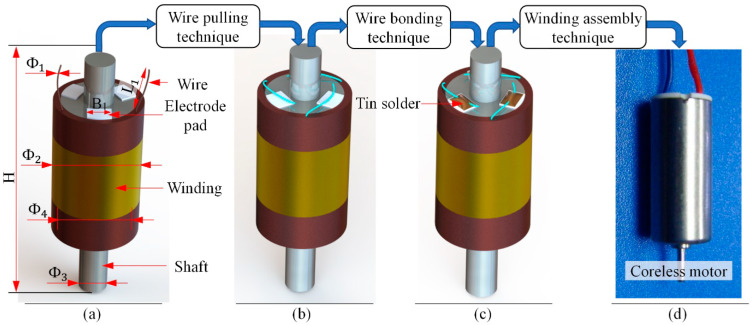
Coreless motor’s winding and its assembly process: (**a**) The winding of coreless motor; (**b**) Electrode wires are dragged and placed on the pads through automated wire bonding technique; (**c**) Electrode wires are soldered through automated wire bonding technique; (**d**) Final coreless motor is assembled through automated winding assembly technique.

The winding of coreless motor is the core component of a coreless motor. Its structure is shown in Figure 1a. It is composed of winding, winding bracket, electrode pad, shaft and electrode wire. The size of each part is shown in Table 1. The diameter of its electrode wire is tens of microns. The electrode wire of the coreless motor winding needs to be welded before it can be assembled into the motor. The whole process is shown in Figure 1. After the winding in Figure 1a is processed by the wire pulling process or wire pulling system, the floating end of the electrode wire is led to the top of the pad, as shown in Figure 1b, and then the floating end of the electrode wire is fixed on the pad through the welding system, as shown in Figure 1c, finally, it is integrated into the coreless motor through the assembly process, as shown in Figure 1d.

**Figure 2:**
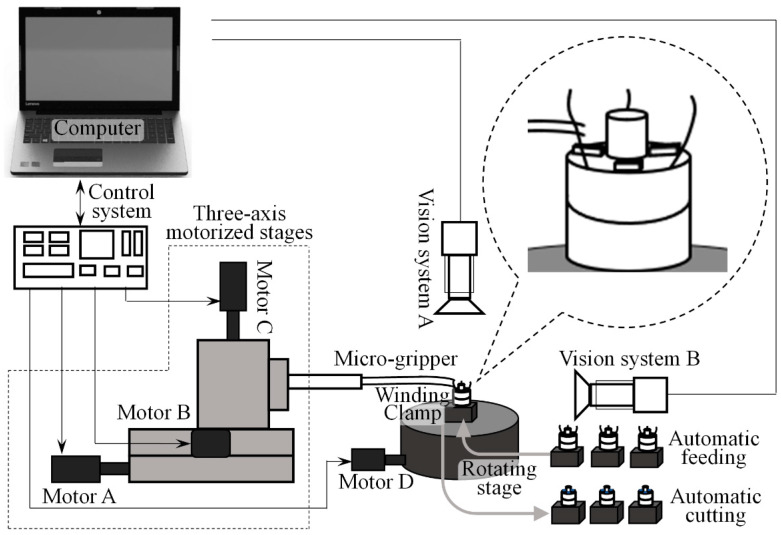
Setup of automated wire pulling system.

At present, most of the wire pulling processes are manually operated. With the help of microscopes, tweezers and other tools, the operator leads the floating end of the electrode wire to the top of the pad by clamping. Because the electrode wire is very thin and bears little external force, manual operation is very difficult. The wire pulling process requires high proficiency of workers, and the efficiency and consistency of manual wire pulling are faultiness. In order to solve this problem, this paper designs the automatic wire pulling micro-manipulation system in Figure 2. The system consists of a micro-gripper system, position and attitude adjustment system, vision system and computer. The micro-gripper system includes a micro-gripper and a drive system, which capture the floating end of the electrode wire by clamping and pulling the floating end to the top of the electrode pad. The position and attitude adjustment system includes three translational degrees of freedom and one rotational degree of freedom. The translational degree of freedom is realized by the motorized stage, which controls the micro-gripper system to move in space. The rotational degree of freedom is used to adjust the angle of the winding and can manipulate multiple electrode wires. The vision system consists of two independent microscopic monocular vision systems, which are composed of a single-cylinder microscope and a camera. The optical zoom ratio is 0.68–4.5×. Vision system A is used to detect the position of the pad, and vision system B is used to track the electrode wire. The whole wire pulling process is controlled by the calculation software, which is responsible for image acquisition, processing and control of the electronic control system. We have studied the control system in Figure 2 [27], and the micro-gripper system is an important research content in the micromanipulation system of wire pulling. In this paper, the structure and opening-closing control of the micro-gripper are designed around the special properties (small diameter, small bearing force, etc.) of the electrode wire in the micromanipulation, and the design of the structure is optimized through simulation analysis.

It can be seen from Table 1 that the diameter and bearing force of the electrode wire are small, the texture is soft, and the shape is a curve of small deformation. In addition, the space around the electrode wire is limited, and it is easy for the micro-gripper to collide and damage the micro-gripper in the process of movement. Therefore, in the process of designing the micro-gripper system, based on these factors we need to consider the material, structure, size, opening-closing displacement, the clamping force of the micro-gripper stress, strain, technology and other properties. Based on the above analysis, the main technical specifications of the micro-gripper system design are given, as shown in Table 2. Monocrystalline silicon is often more suitable for micro-electro-mechanical systems (MEMS), which is very suitable for processing micro size structures, and has very good mechanical properties in micro-scale conditions. The clamping method adopts envelope type, which produces a hexagon structure after closing, and can pull the wire after holding the root of the wire. The maximum computational load is designed to be 0.5 N (the data is obtained by analyzing the experimental measurement results in Section 3.1). In Figure 1a, the plane manipulation length around a single wire is about 1 mm, so the maximum characteristic size of the micro-gripper’s tip is not more than 0.3 mm. In the working process of the micro-gripper, it is required that the opening-closing distance can be adjusted dynamically, so the motorized stage is used to control the opening-closing.

### 2.2. Opening-Closing Control System

**Figure 3:**
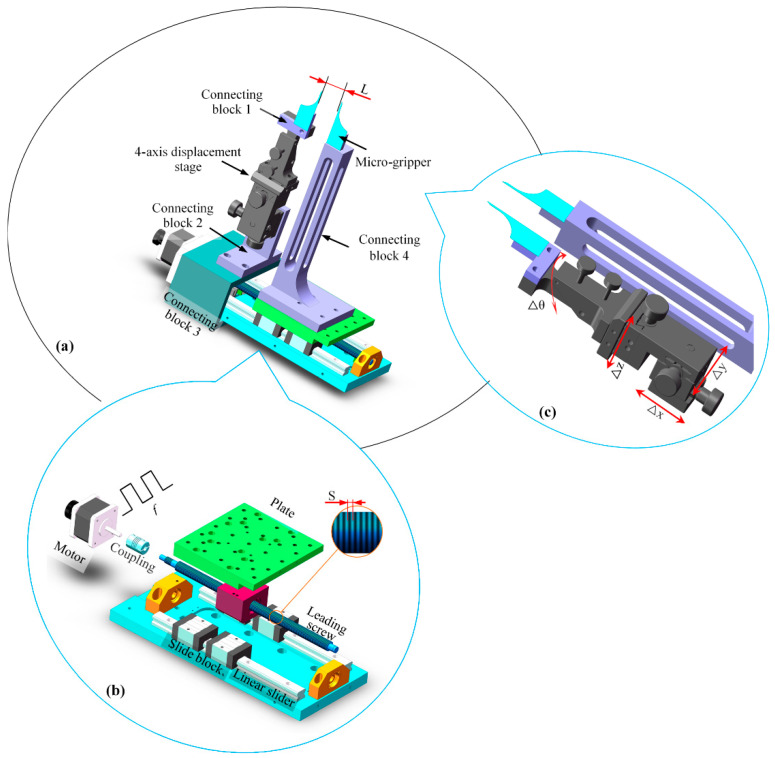
Setup of micro-gripper: (**a**) Three-dimensional model of micro-gripper; (**b**) Local motion system for opening-closing control; (**c**) Local setup of micro-gripper.

The three-dimensional model of the micro-gripper system is shown in Figure 3a, which adopts the structure of double micro-gripper arms, including drive mechanism, micro-gripper arm, precision alignment mechanism, and micro-gripper tip. The four mechanisms are connected by the assembly. One micro-gripper’s tip is installed on a precision alignment mechanism, which is connected with the static arm, which is fixed on the base, the other micro-gripper’s tip is fixed on the boom, which is installed on the slider of the drive mechanism and can move with the slider. The driving force is generated by the driving mechanism, which makes the micro-gripper arm produce an opening-closing motion.

#### 2.2.1. Opening-Closing Control Principle

Figure 3b is an exploded view of the driving mechanism, which is composed of a stepping motor, double linear slider, leading screw and slider block. The rotation of the stepper motor is transformed into the translational movement of the slide block through the leading screw drive. The static arm is installed on a base and does not participate in the movement. The boom is installed on the slide block and can move horizontally with the slide block. The left and right micro-gripper arms can do the opening and closing movement through the motor rotation. In the process of movement, the screw moves synchronously with the motor through the coupling. At the same time, the screw and the leading screw interact to realize the linear movement of the screw. Because the micro-gripper arm and the micro-gripper are connected to the translation table by bolts, the translation table is fixed with the screw nut. Through the motor driving mode, the rotary motion is transformed into linear motion, so as to realize the movement of the boom, while the position of the static arm remains unchanged. Through the relative movement of the boom and the static arm, the micro-gripper tip can produce the opening and closing movement.

Because the transverse dimension of the micro-gripper’s tip is in the order of hundreds of microns, the two micro-gripper’s tips need to be precisely aligned before use, which is realized by a precision alignment mechanism with four degrees of freedom. As shown in Figure 3c, the precision alignment mechanism is installed on one of the micro-grippers, which can realize the translation in three directions (the maximum translation distance is less than 3 mm) and the pitch adjustment in one direction (the maximum pitch angle is 5°). Both of them are slightly adjusted, so that the two pincers are precisely aligned.

#### 2.2.2. Opening-Closing Control

The opening-closing amount and opening-closing speed of the micro-gripper are realized by the rotation angle and rotation speed of the stepper motor, which are controlled by the frequency and number of square wave pulses that control the rotation of the motor.

Considering the load factor, the torque of the stepper motor should meet the demand of the actual load. Under the condition of no overload, the opening-closing displacement and speed of the micro-gripper only depend on the frequency and number of pulses of the pulse signal. Set the stepping angle of the stepping motor as α, the lead of the leading screw is S, the subdivision of the stepper motor driver is N, and the total number of steps is *N_α_ =* 360·*N*/*α*, and the translation distance produced by each step *L_α_ = S/N_α_*. Then, the opening-closing displacement L and the opening-closing speed *v* of the micro-gripper are calculated according to the following formula:

(1) $$\left\{ \begin{array}{l} L=L_{\alpha }\times n\\ v=S\times \frac{f}{N_{\alpha }} \end{array}\right.$$

where *n* is the number of pulses to control the motor and *f* is the pulse frequency.

In Table 3, the estimated results of the opening-closing displacement *L* and the opening-closing speed *v* of the micro-gripper under several conditions are given α = 8°, lead *S* = 4 mm, and subdivision N is 8, it can be seen from Table 3 that the speed V is only related to the frequency, the higher the frequency is, the higher the speed is. When the frequency is set to 1080 Hz, its speed can reach 2.7 mm/s, and its displacement is only related to the number of input pulses. The larger the number of input pulses is, the greater the bit shift of its movement is.

### 2.3. Micro-Gripper’s Tip Design

The micro-gripper’s tip is an important part of the micro-gripper. Its design should meet the property requirements of the electrode wire and the limited conditions of the micro-manipulation space given in Section 2.1. It mainly includes three parts: structure design, technical parameter design, and performance simulation.

#### 2.3.1. Structural Design

**Figure 4:**
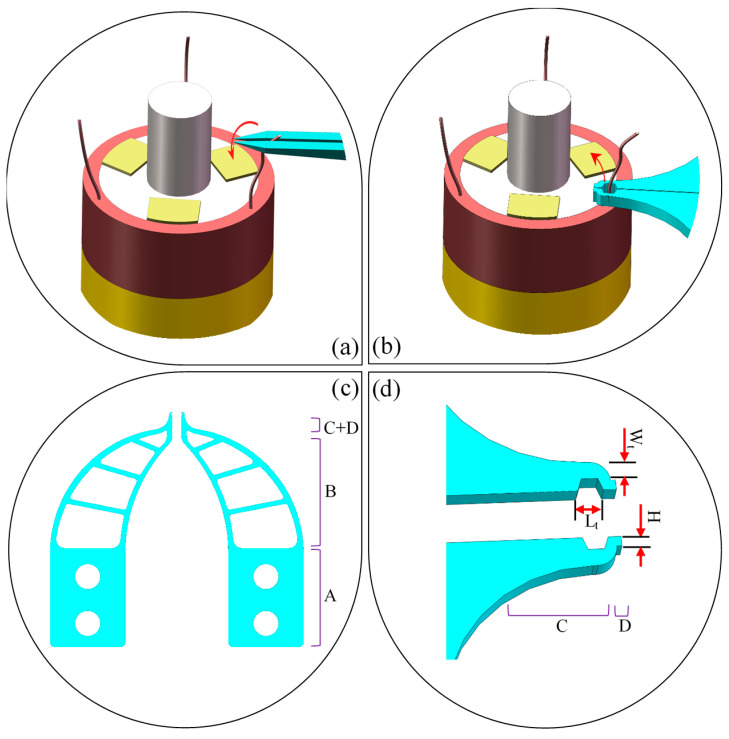
Design of clamping strategy for thin electrode wires: (**a**) Direct clamping strategy and tip of its corresponding micro-gripper; (**b**) Enveloping strategy and its corresponding micro-gripper, where ‘A’ is called the assembly region, ‘B’ is called the elastic-beam region, ‘C’ is called the tip-enveloped region, ‘D’ is called tip-protected region; (**c**) Model of enveloping-type micro-gripper’s tip; (**d**) The enveloped local micro-gripper’s tip containing the regions C and D.

Figure 4a is a clamping strategy of clamping type micro-gripper tip. The shape of the micro-gripper tip is straight and not specially designed. After the micro-gripper tip is closed, the floating end of the electrode wire is clamped, and then the floating end is pulled to the top of the pad for release. Figure 4b is a clamping strategy for enveloping the micro-gripper tip. After the micro-gripper tip is closed, it forms a closed polygon or circular shape, which can be encircled from the root of the electrode wire, and then the micro-gripper tip moves to the top of the pad. Because the electrode wire is a flexible body, it deforms with the movement of the micro-gripper tip and is released at the top of the pad. Due to the deformation of the electrode wire and the unfixed position of the suspension end, the position of the suspension end needs to be calculated by the stereo vision system before the clamping micro-gripper tip clamps the suspension end, which is often uncertain. Therefore, when the clamping micro-gripper tip in Figure 4a is used to manage the flexible electrode wire, there will be instability. The position of the root of the electrode wire is relatively fixed and will not fluctuate greatly with the deformation of the wire. Therefore, it is easier to achieve accurate clamping by using the enveloping micro-gripper tip in Figure 4b. By using the flexible deformation of the electrode wire, the wire can also be guided to the pad position. Compared with the clamping micro-gripper tip, the enveloping micro-gripper tip is more suitable for the wire pulling of the flexible electrode wire, so this paper adopts an envelope structure design.

Figure 4c is a model diagram of the micro-gripper’s tip, which adopts a dual-arm symmetrical structure. Each micro-gripper arm includes four regions: assembly region, elastic-beam region, tip-envelope region, and tip-protection region. The assembly components are used for fixing and connecting. Two through-holes are designed in the assembly region to fix the micro-gripper tip with the micro-gripper arm or precision alignment mechanism in Figure 3c. The elastic-beam region connects the tip-envelope region and the assembly area. Because the size of the tip-envelope region is in the order of several hundred microns, it cannot be directly fixed with other parts. In addition, the maximum stress of the micro-gripper tip usually occurs in the tip-envelope region during the clamping process, and the flexible deformation of the elastic-beam region can provide a buffer to protect the tip envelope region from hard damage. After the micro-gripper tip-envelope region is closed, it is a closed polygonal structure. This design can not only meet the needs of space and capturing wires, but also maximize the material retention and increase its strength. The 3D structure of the enlarged tip envelope area is shown in Figure 4d. In addition, in Figure 4d, a square tip-protection region is designed at the top of the tip-envelope region to prevent direct damage to the tip-envelope region when colliding with the shaft of winding or other parts.

#### 2.3.2. Technical Parameter

In the process of designing the micro-gripper tip structure in Figure 4c, the material, processing technology, and geometric dimension are mainly considered. Materials have a great influence on the mechanical properties of the micro-gripper tip. The optional materials include steel, iron and silicon. If steel, iron and other materials are used, the micro-gripper tip is usually manufactured by precision machining. However, because the local size of the micro-gripper tip is in the order of tens to hundreds of microns, the precision machining process is restricted by the machining accuracy, and it is difficult to ensure the integrity of the shape. The special envelope shape of the tip cannot be machined, or the machining accuracy cannot meet the predetermined requirements. Silicon materials are widely used in the field of MEMS. Small size microstructures are fabricated by photolithography, corrosion, etching and other processes, which have great advantages in shape integrity and machining accuracy. Table 4 is the comparison of performance parameters of steel, iron and silicon materials. It can be seen from the table that although the density of silicon material is small, its tensile strength and hardness are much higher than stainless steel and other materials. The beam made of monocrystalline silicon has good elasticity and no plastic deformation, and its reliability is better than the metal microstructure with the same size and structure. Based on this, this paper uses silicon material to make the tip part of the micro-gripper, selects 100 single crystal silicon, and processes the microstructure at the tip by the deep reactive ion etching process. The etching depth is 0.1–0.4 mm. The main process includes photolithography, deep silicon etching and cleaning.

**Figure 5:**
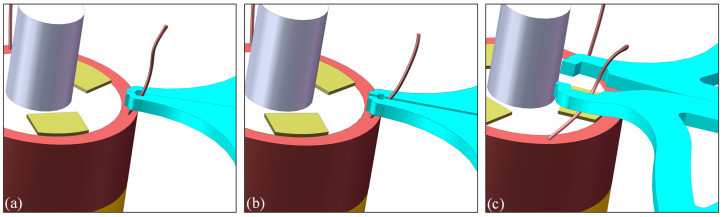
Analysis for the influence of dimension of the enveloped shape on clamping wires: (**a**) The wire is clamped accurately by the tip with proper size; (**b**) Incorrect clamping is due to the small size of the enveloped shape, and the wire is located on the outside of the enveloped shape; (**c**) Due to the large size of the enveloped shape, the tip collides with the shaft, which may damage the tip.

Figure 4 shows the main geometric dimensions of the micro-gripper tip design. The length L and width W of the micro-gripper tip are designed to be 25 mm and 13 mm, respectively, which is convenient for fixing with other parts. The shape of the tip-envelope region is designed as a hexagon, which can completely surround the wire when it is closed, and it is easy to process and keep the designed shape. When the tip-envelope region is closed, the hexagonal structure is shown in Figure 5a, which includes three-dimension parameters: *L_t_*, *U_t_* and *θ*. Because the diameter *Φ* of the electrode wire ranges from 0.05 mm to 0.07 mm, the value of *U_t_* and *L_t_* should be at least greater than 0.07 mm to capture the electrode wire completely. In the process of gradual closure of the tip-envelope region, when the value of *L_t_* is smaller, once the wire is deformed or tilted, the closed hexagon will not be able to completely surround the wire, as shown in Figure 5b. From this point of view, the larger the value of *L_t_*, the more accurate it is to capture the wire. However, when the value of *L_t_* is much larger than the diameter of the electrode wire, the size of the tip-envelope region will increase, and the probability of collision with other parts will increase during the movement, as shown in Figure 5c. From this perspective, the smaller the value of *L_t_*, the more useful it is to reduce the probability of damage to the micro-gripper tip. Considering these factors, the value of *L_t_* is limited to [2Φ, 4Φ]. The value of *L_t_* is limited in the range of [0.14 mm, 0.28 mm]. In the process of capturing the electrode leads, *U_t_* and *θ* have less influence on the rationing, setting *θ* to 30° and the range of *U_t_* values to [2Φ, 3Φ], that the value of *U_t_* is limited to the interval [0.14 mm, 0.21 mm].

The thickness parameter *H* of the micro-gripper tip depends on the thickness of the silicon wafer, choosing sizes such as 0.1 mm, 0.2 mm, 0.3 mm and 0.4 mm. The greater the value of *W_t_*, the more external forces (such as the reaction force of electrode wire, the force applied by other parts in the process of collision force, etc.) can be borne in the envelope region, which reduces the deformation of the envelope in the process of wire pulling, but increases the size of the tip-envelope region, and increases the probability of collision and failure. Based on the above considerations, the value of *W_t_* is limited to the range [*Φ*, 4*Φ*], i.e., *W_t_* is limited to the range [0.07 mm, 0.28 mm]. The frame dimensions *C*1 and *C*2 of the tip-protected region are set to [*Φ*, 2*Φ*]. That is, in the range of [0.07 mm, 0.14 mm]. In conclusion, the main geometric parameters of the micro-gripper tip in Figure 4c are shown in Table 5.

#### 2.3.3. Simulation Analysis of Mechanical Properties

**Figure 6:**
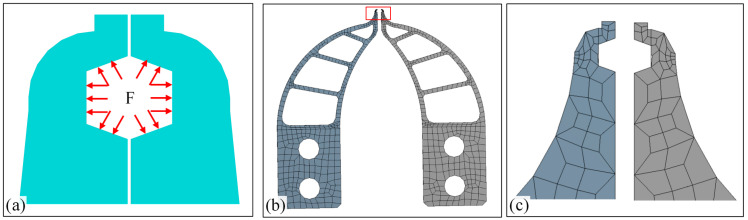
Force and finite element model for micro-gripper’s tip: (**a**) Force model, where *F* represents the force exerted by the wire on the inner edge of the enveloped region; (**b**) Finite element model of micro-gripper’s tip; (**c**) Locally enlarged finite element model corresponding to the area marked by a red box in subfigure (**b**).

The tip-envelope region captures the electrode wire and in the later movement process, it will be subject to the reaction force *F* of the electrode wire, as shown in Figure 6a. In the whole micro-gripper movement, the force direction of the micro-gripper is not fixed, and all surfaces of the whole envelope shape may be contacted. Therefore, the force direction in the simulation is analyzed as shown in Figure 6a. According to the experiment in Section 3.1, the limit force exerted by the conductor is 0.3 N. From the perspective of design reliability, the force applied in the simulation process should be greater than 0.3 N, and the maximum value of *F* should be set to 0.5 N. The mechanical simulation of the micro-gripper tip is carried out to analyze whether the structure and geometric dimensions of the micro-gripper tip in Figure 4c meet the needs of stiffness and deformation. The main mechanical indexes of simulation are stiffness index and stress index. The stiffness index is a physical quantity to evaluate the deformation (elastic deformation and plastic deformation) of the micro-gripper tip. Under the action of force *F*, if the stiffness index does not meet the requirements, the tip-envelope region may produce large deformation, which may affect the clamping effect of the electrode wire and may also damage the micro-gripper tip. The stress index is a physical quantity to evaluate the strength of the micro-gripper tip. If the maximum stress exceeds the strength limit, the micro-gripper tip will be damaged. In the ANSYS Workbench 19.0 software, the mechanical simulation of the micro-gripper tip in Figure 4c is carried out, and the finite element model is shown in Figure 6b. The whole process mainly includes model import, material attribute definition, mesh generation, fixation and load, solution, etc. The model is imported into ANSYS Workbench 19.0, and the properties of SI material are defined. The elastic modulus of SI material is 112.4 Gpa, the mass density is 2330 kg/m^3^, and the Poisson’s ratio is 0.28. The hexahedron body method is selected for mesh generation. The parts are fixed and constrained by bolt connection in the assembly area, and the tip-envelope region is loaded. The possible stress forms are shown in Figure 6a. There are three main stress directions in the movement process. Finally, the specific results of the finite element are obtained by solving the problem.

In this section, the stress distribution of the micro-gripper tip is simulated and analyzed. In the process of simulation, the thickness parameter *H* of the micro-gripper tip and the transverse width parameter *W_t_* of the tip-envelope region are changed to take different values. The structural models with different parameters are established in SolidWorks software and imported into ANSYS Workbench 19.0 software for simulation. Firstly, a set of parameters of *H* and *W_t_* are given, and the simulation is carried out under the parameters to analyze the stress distribution and deformation of the micro-gripper tip in different regions. Then, keep *H* or *W*_t_ unchanged, and gradually increase the value of *Wt* or *H* from the reference value to obtain the maximum stress change curve and maximum deformation change curve of the tip-envelope region in the process of parameter change. Through the analysis of these curves, the optimal values of *H* and *W_t_* are determined.

**Figure 7:**
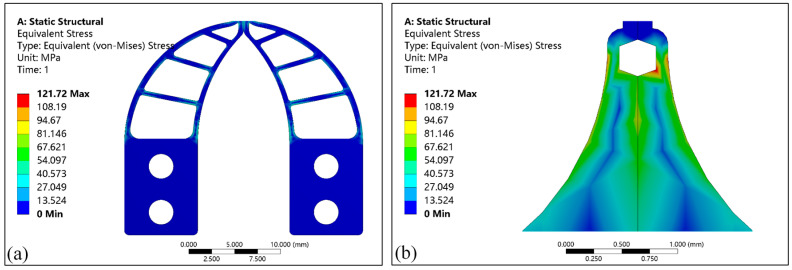
The simulation results of stress distribution for micro-gripper’s tip: (**a**) The stress distribution in the assembly and in elastic-beam regions; (**b**) The stress distribution in the tip-enveloped and tip-protected regions.

Let *H* = 0.2 mm, *W_t_* = 0.08 mm, and simulate under this condition to analyze the stress distribution and deformation distribution of the micro-gripper tip. Figure 7a is the stress distribution of the tip assembly region and elastic-beam region, and Figure 7b is the stress distribution diagram of the tip-envelope region and the tip-protected region. It can be seen from Figure 7a that the stress in different regions is different, and the maximum stress appears in the elastic-beam, with a value of 53.905 MPa. It can be seen from Figure 7b, the maximum stress occurs in the tip-envelope region, with a value of 121.72 MPa. With the continuous change of the model size, the maximum stress value will change, but the maximum stress area of each region will not change.

**Figure 8:**
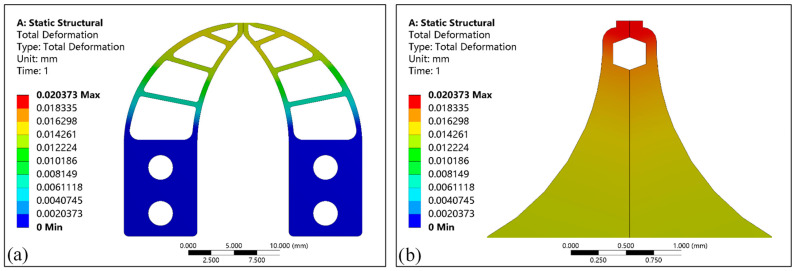
The simulation results of the deformation of the micro-gripper’s tip. (**a**) The deformation distribution in the assembly and in elastic-beam regions; (**b**) The deformation distribution in the tip-enveloped and tip-protected regions.

Figure 8a shows the deformation distribution of the micro-gripper’s tip assembly region and the elastic-beam region, and Figure 8b shows the deformation distribution of the tip-envelope region and the tip-protected region. From Figure 8a,b, it can be seen that the deformation in different regions is different, and the maximum deformation occurs in the tip region, and its value is 20.373 μm. With the continuous change of width and thickness parameters, the deformation will also show regular changes.

**Figure 9:**
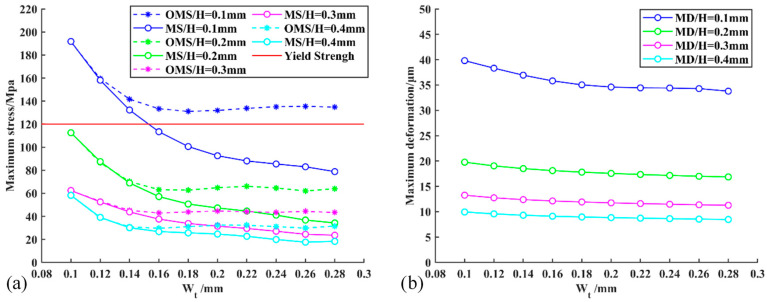
The relationship curves between maximum stress, maximum deformation and the geometric parameters *H* and *W_t_* of the micro-gripper’s tip are obtained by simulation, where *H* and *W_t_* have been shown in Figure 4c. (**a**) The relationship curves related to maximum stress, *H* and *W_t_*; (**b**) The relationship curves related to maximum deformation, *H* and *W_t_*. Where MS = Maximum stress, OMS = Overall Maximum stress, MD = Maximum deformation.

The thickness parameter *H* of the silicon wafer is set to four kinds of values: 0.1 mm, 0.2 mm, 0.3 mm and 0.4 mm. These kinds of thickness silicon wafers are very common. Then the micro-gripper tip model with different *W_t_* values is designed on each kind of thickness silicon wafer. The value range of *W_t_* is [0.07 mm, 0.28 mm]. In this range, the value of *W_t_* is selected, and the value spacing is set to 0.02 mm. There are 10 values of *W_t_*, for each pair of *H*-*W_t_* values corresponding to the tip model, and the maximum stress and maximum deformation in the tip-envelope region of each model tip are calculated. Figure 9a shows the maximum stress distribution obtained under each *H*-*W_t_* model. Curve fitting is performed on these data to obtain a continuous *W_t_* maximum stress curve. It can be seen from the figure that the solid line is the stress curve at the tip of the micro-gripper, and the dotted line is the stress curve of the whole micro-gripper structure. It can be seen that with the increasing width parameter *W_t_*, the stress gradually decreases, and with the increasing thickness, the stress also decreases. Figure 9b shows the maximum deformation distribution under each *H-W_t_* model. Curve fitting is performed on these data to obtain the continuous maximum deformation curve under *H-W_t_*. It can be seen that with the increasing width parameter *W_t_* and thickness parameter *H*, the deformation also presents a gradually decreasing trend. Considering the stability and reliability in the actual operation process, the maximum deformation curve under *H-W_t_* is obtained. Under the condition that the design meets the stress requirements, the ideal model is 0.2 mm in thickness and 0.18 mm in width.

### 2.4. Clamping Traction Strategy

**Figure 10:**
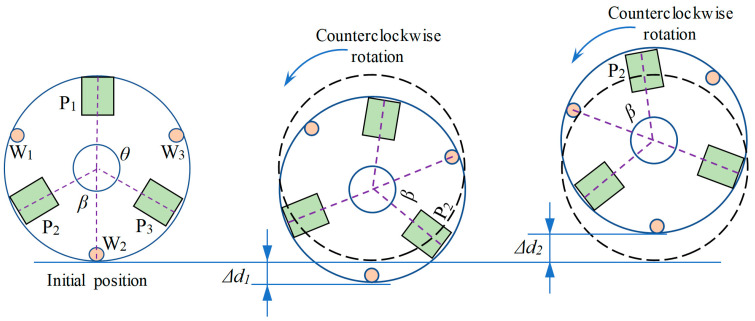
Diagram of the rotating eccentricity of the coreless motor winding.

The coreless motor winding relies on a rotary translation table to rotate the winding during clamping and traction, and due to machining and assembly errors, eccentricity can occur during the rotation process. Figure 10 shows a schematic diagram of the rotational eccentricity of the coreless motor winding, from which it can be seen that at the initial moment the outer edge of the coreless motor winding is tangent to the reference position, and as the coreless motor winding rotates, it is offset by a certain amount, e.g., Δd1, Δd2. To solve this problem, a microscopic vision system was used for detection compensation. Using image processing techniques, the outer contour of the coreless motor winding is identified and then a circle is fitted to the outer contour to extract the position of the center of the circle.

**Figure 11:**
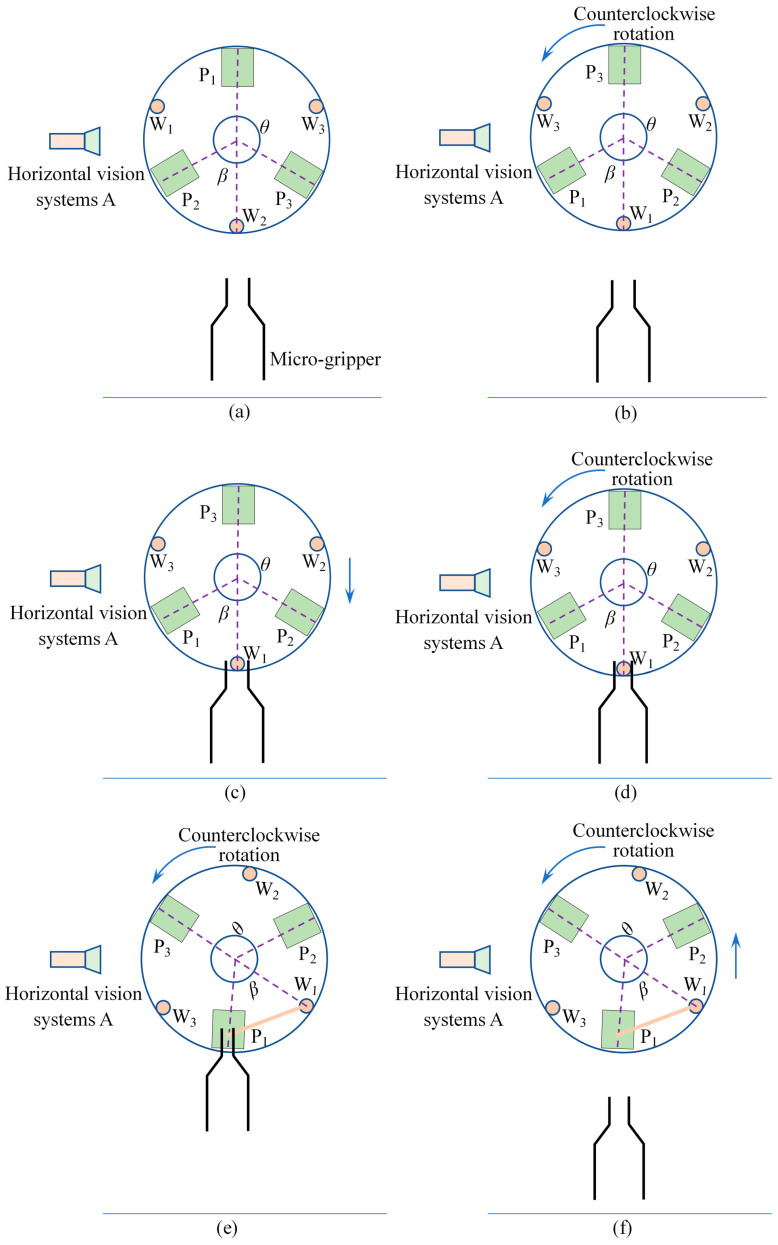
Electrode wire clamping traction strategy: (**a**) Electrode wire and pad positioning inspection; (**b**) Turn counterclockwise to the first wire position; (**c**) Electrode wire moved to clamp position; (**d**) Micro-gripper closure; (**e**) Electrode wire pulling to pad position; (**f**) Micro-gripper opens and returns to the initial position.

Figure 10 shows that the three electrode wires and three electrode pads are distributed at the top of the coreless motor winding and that they are not equally spaced, with different angles between them. We use a microscopic vision system for detecting the target position, which consists of a horizontal vision system A and a vertical vision system B. The horizontal vision system A is used to detect the position of the electrode wires and the vertical vision system B is used to detect the electrode pads, as well as the eccentric position. Figure 11a shows the inspection of the electrode wire and electrode pad positions and the eccentric position. During the inspection phase, the winding is rotated at a constant speed of V1 using a rotary table, the electrode wire position is detected by the horizontal vision system A and the position information is recorded, and the vertical vision system B detects the position of the outer circle of the winding and records the eccentric amount information by calculation. Figure 11b shows the rotary table rotating counterclockwise to the first wire position at speed V2 (V2 > V1) in preparation for clamping traction when the inspection is complete. Figure 11c shows that after determining the relative position of the micro-gripper to the wire, the micro-gripper is moved up and down to a position 5 mm from the root, considering that the root position of the electrode wire is more fixed and convenient for positioning. Figure 11d shows the micro-gripper being closed and the electrode wires wrapped. Figure 11e shows the electrode wires being pulled to the pad position by the movement and rotation of the winding. Figure 11f shows the micro-gripper opening after the traction manipulation has been completed, retreating to the initial position to wait for the next wire to be manipulated and repeating the above steps until traction is complete.

## 3. Results

**Figure 12:**
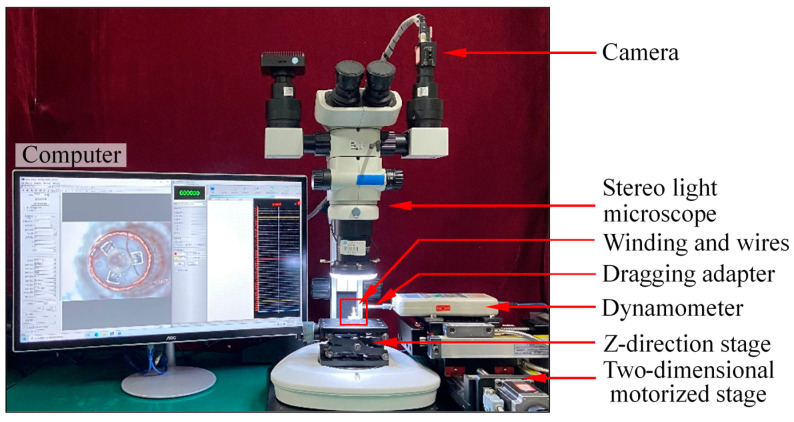
Setup of experimental system.

In this section, the maximum force of the electrode wire and the fatigue degree of the micro-gripper tip are estimated through experiments, and the mechanical properties of the micro-gripper tip are analyzed. In addition, the clamping effect of the micro-gripper is verified through experiments. Figure 12 is an experimental system for testing stress and fatigue. Olympus stereo light microscope is used to form a micro vision system to observe the pulling process in the experiment. The model of the stereo microscope is SZX-7. A camera is installed on the imaging surface of the microscope, and the resolution of the camera is 1280 × 1024 pixels. A displacement table is placed in the object space of the stereo light microscope. The winding is inserted on the base and fixed on the table of the displacement table together with the base. The displacement table can adjust the displacement along the Z direction. Use a dynamometer to pull the electrode wire or micro-gripper tip to measure the maximum bearing force of the electrode wire. The dynamometer is a high-precision digital push-pull meter with a range of 0~5 N, an accuracy of 1% of the measured value, and a sampling rate of up to 1000 times/s. The dynamometer is fixed on a set of the two-dimensional motorized stage, which can move along the *x*-axis or *y*-axis direction. The repeated positioning accuracy of the two-dimensional motorized stage is 1 μm. When the two-dimensional motorized stage moves to the right, pull the electrode wires or micro-gripper tips. When they break, the measured value of the dynamometer is taken as their maximum bearing force.

### 3.1. Measurement of Maximum Bearing Force of Electrode Wire

**Figure 13:**
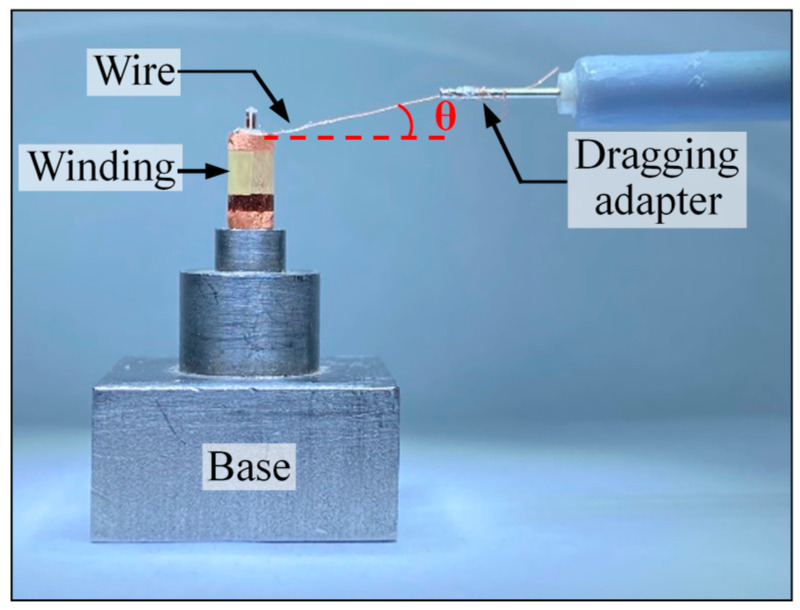
The partial enlargement of the area is marked by a red box in Figure 10, and it shows the rule of measuring the maximum pulling force on wires.

The measurement principle of the maximum bearing force of the electrode wire is shown in Figure 13. A connector for connecting the electrode wire and the dynamometer is designed, which is made by 3D printing technology. After the wire is straightened, the angle between the straight line and the horizontal plane is *θ*, the size of the angle *θ* will affect the result of the bearing force measurement. The size of the angle is divided into three measuring intervals: 0–5°, 10–15°, and 40–50°. By adjusting the height of the Z-direction stage, the electrode wire can be tied to a certain angle interval, and then the position of the XY motorized stage can be adjusted to move the connector to the right side. When the wire breaks or cracks at the root, the value of the dynamometer is read. This value is used as the measurement value of the maximum bearing force of the electrode wire. The length of the electrode wire is 1.5~2 mm. The electrode wire is extended by adhesive and then connected with the dragging adapter.

**Figure 14:**
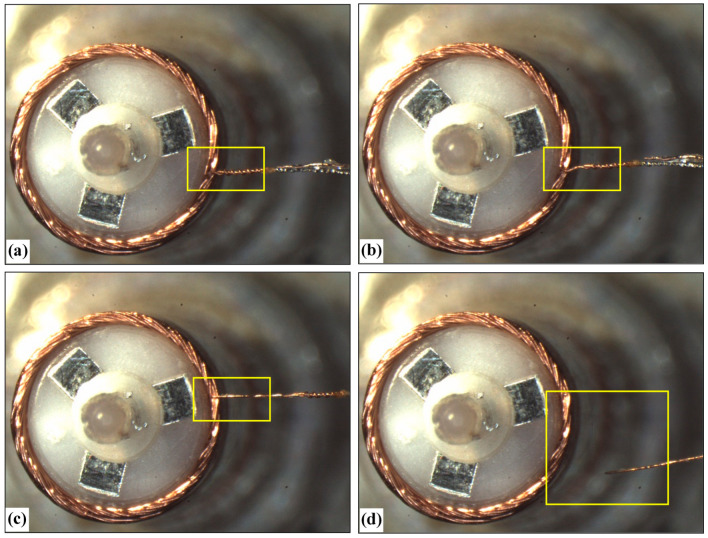
The measurement process of the maximum pulling force on wires: (**a**) Status before applying pulling force; (**b**) Status in applying pulling force; (**c**) The shape of the wire begins to as pulling force increases; (**d**) The wire is broken under large pulling force.

In the experiment, 6 windings were selected as experimental samples, each sample contained 3 electrode wires, a total of 18 wires. Two coils with a total of 6 wires are used in each angle interval of *θ*. Figure 14 is the four intermediate-links in the process of bearing force measurement. Before applying the tension, as shown in Figure 14a, the tension is applied, as shown in Figure 14b, the wire is disconnected, as shown in Figure 14c, and the wire is broken, as shown in Figure 14d.

Table 6 shows the measurement results of the maximum bearing force of the electrode wire. In the range of *θ* of 0–5°, the maximum bearing force is 0.29 N. In the range of *θ* of 10–15°, the maximum bearing force is 0.275 N; in the range of *θ* of 40–50°, the maximum bearing force is 0.229 N. To sum up, the maximum bearing force of electrode wire is less than 0.3 N, less than 0.5 N in Section 2.3.3, which can ensure the reliability of micro-gripper tip design.

### 3.2. Stiffness Analysis of Tong Tip

**Figure 15:**
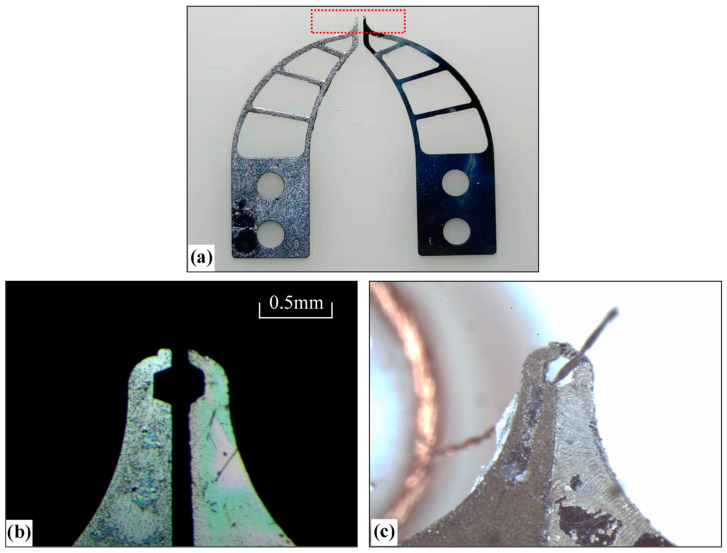
Picture of silicon micro-gripper’s tip: (**a**) The whole of micro-gripper’s tip; (**b**) Local picture in the tip-enveloped and tip-protected regions related to the red box area in (**a**); (**c**) Picture of clamping the wire.

Figure 15a shows the micro-gripper tip structure fabricated on a 0.2 mm thick silicon wafer, Figure 15b shows the image of the tip region photographed under the microscope, and Figure 15c shows the state of encircling the wire when the tip-envelope region is closed. It can be seen from the figure that the shape of each region of the micro-gripper tip is maintained well, and it can encircle the wire, which can achieve the expected function.

**Figure 16:**
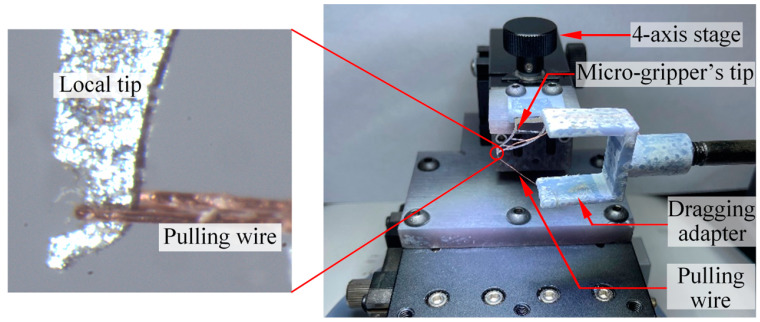
Setup of stiffness measurement system for micro-gripper’s tip.

The micro-gripper tip in Figure 15 is used to analyze the stiffness index. The principle of the experiment is shown in Figure 16. The copper wire is used to bind the tip-envelope region, and then the copper wire is fixed to the dragging adapter. The dragging adapter is connected with the dynamometer in Figure 12. The system is used to pull the tip-envelope region in Figure 12, apply different pulling forces, and take the image of the tip-envelope region through the camera in Figure 12. The deformation of the tip-envelope region in the tensile direction is measured in the image. In the experiment, the micro-gripper tip is fixed on a set of three-dimensional micro displacement platforms, which can adjust the position of the micro-gripper tip in a small range. In the process of pulling the tip-envelope region, the force application range of the dynamometer is controlled within 0~0.6 N, and the tension is gradually increased. The increment of the tension is 0.1 N. The images are taken, and the deformation is measured under 0 N, 0.1 N, 0.2 N, 0.3 N, 0.4 N, 0.5 N and 0.6 N, respectively.

**Figure 17:**
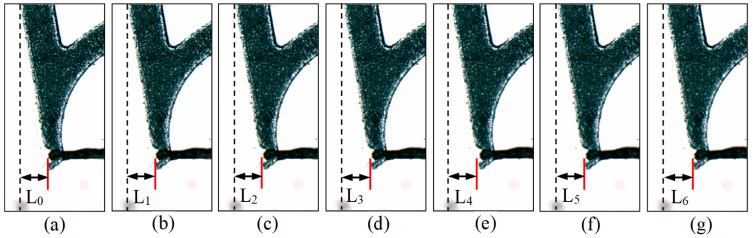
Results of the deformation measurement in images under different pulling force for micro-gripper’s tip. (*F*): (**a**) Deformation under *F* = 0 N; (**b**) Deformation under *F* = 0.1 N; (**c**) Deformation under *F* = 0.2 N; (**d**) Deformation under *F* = 0.3 N; (**e**) Deformation under *F* = 0.4 N; (**f**) Deformation under *F* = 0.5 N; (**g**) Deformation under *F* = 0.6 N.

Figure 17a–e are images of the tip-envelope region captured under different tension conditions. In the image, the deformation *L*_0_–*L*_6_ of the tip-envelope region is measured with a fixed position as the reference point. In the process of measurement, the micro vision system is calibrated, and the space distance of a single pixel in the image is 1.72 μm.

Table 7 is the measurement results of the deformation of the tip-envelope region under different tensile forces. It can be seen from Table 7 that the deformation increases with the increasing tensile force. According to the experiment in Section 3.1, the maximum force exerted by the electrode wire on the tip-envelope region will not exceed 0.3 N during wire pulling. It can be seen from Table 5 that when the force is 0.3 N, the deformation of the tip-envelope region is 27.52 μm. In this case, the electrode wire will not be separated from the tip-envelope region, and the wire pulling effect will not be affected. On the other hand, when the tensile force reaches 0.5 N, the deformation of the tip-envelope region is 34.4 μm, approximately equal to *Φ*/2. After the load is removed, it can return to the initial position, indicating that its stiffness meets the requirements. In the simulation of Section 2.3.3, when the applied force is 0.5 N, the deformation of the envelope area is 17.79 μm. The difference between simulation results and experimental results is 16.61 μm. There are two main reasons for this error: (1) In the experiment, the applied load is a concentrated force located at a certain position in the tip-envelope region, and the acting point of the force is more inclined to the top of the tip-envelope region. (2) In the experiment, there are assembly errors and measurement errors, which will also affect the measurement results. When the tensile force reaches 0.6 N (which has exceeded the maximum applied force of 0.5 N in Section 2.3.3), the deformation of the tip-envelope region is 43.0 μm. It is closer to the diameter of the electrode wire, and the deformation is very obvious.

### 3.3. Micro Clamping Operation

The whole automatic wire pull robot system includes a vision system, micro-gripper system, motion system, and so on. Among them, the vision system is used to realize the identification, positioning and eccentricity detection of the electrode wire, the micro-gripper system is used for the clamping and traction manipulation of the electrode wire, and the motion system is used to realize the movement and rotation of the winding of the coreless motor in space. In the operation process, the vision system first identifies and locates the electrodes and transmits the position information to the PC, which moves the hollow cup rotor coil through the control box and the motion system and operates the wire traction with the micro-gripper system.

**Figure 18:**
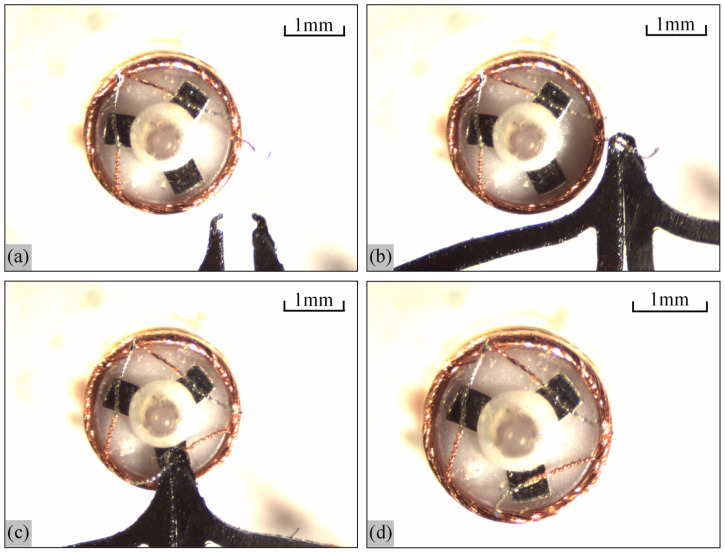
A process of clamping a wire with micro-gripper: (**a**) Initial positions of micro-gripper and wire; (**b**) The status that micro-gripper captures the wire; (**c**) The status that micro-gripper carries the wire over the pad; (**d**) The status that micro-gripper releases the wire.

The micro-gripper tip in Figure 15 is assembled with the motion control system in Figure 3 and then integrated into the system in Figure 2 to test the clamping performance of the micro-gripper. Figure 18 shows the process of the micro-gripper tip clamping the wire under the micro vision system. Figure 18a shows the state of the micro-gripper when it is open, and then the micro-gripper begins to move along the Y direction and closes when it reaches the root of the electrode wire, as shown in Figure 18b; after capturing the wire, the micro-gripper starts to move to the pad position and reaches the top of the pad, as shown in Figure 18c. Release the electrode wire here and exit the field of view of the microscope. At this time, the electrode wire is already at the top of the wire, and the wire management process is completed, as shown in Figure 18d. It can be seen from Figure 18 that the micro-gripper system designed in this paper can pull the electrode wire to the top of the pad, which can realize the line management function.

## 4. Discussion

**Figure 19:**
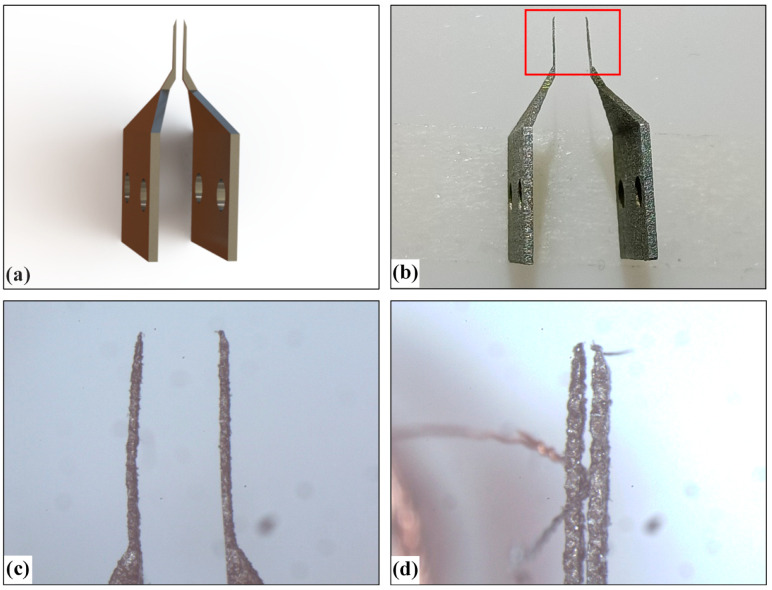
Direct-clamping-type micro-gripper made of iron: (**a**) 3D model of micro-gripper; (**b**) Picture of micro-gripper; (**c**) Picture of local micro-gripper’s tip corresponding to red box area in (**b**); (**d**) The status that micro-gripper captures a wire.

**Figure 20:**
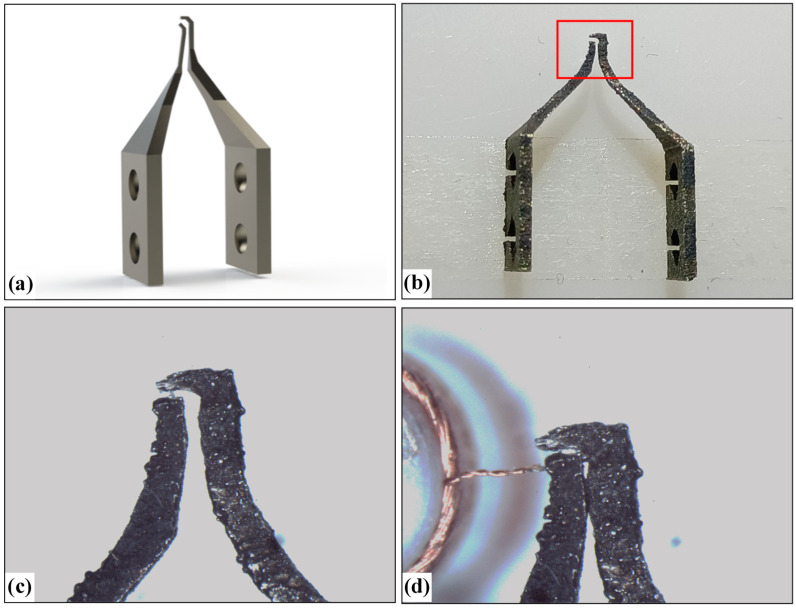
Enveloping-type micro-gripper made of steel: (**a**) 3D model of micro-gripper; (**b**) Picture of micro-gripper; (**c**) Picture of local micro-gripper’s tip corresponding to red box area in (**b**); (**d**) The status that micro-gripper captures a wire.

To compare the performance of silicon micro-gripper with other materials, we designed two kinds of clamping type micro-grippers with steel materials, as shown in Figure 19 and Figure 20, which are made by machining.

Figure 19a is the model of the first straight arm type micro-gripper, Figure 19b is the photo of the machined micro-gripper, Figure 19c is the microscopic image of the microgripper tip structure, and Figure 19d is the state when the electrode wire is clamped by the micro-gripper. It can be seen from Figure 19c that the surface of the straight arm micro-gripper made by machining is rough, which is not as smooth as that of the silicon microgripper in Figure 15, and the machining quality of the shape is also quite different. When the width of the micro-gripper tip is small, the rigidity of the straight arm micro-gripper tip is weak, and it is easy to have large deformation, as can be seen from Figure 19d. In addition, as shown in Figure 19d, the clamping and traction experiments were carried out, through experimental analysis we found that because of this micro-gripper in the clamping and traction manipulation, the suspended end of the electrode wire needs to be clamped, but the position of the suspended end is more arbitrary, it is difficult to position it, if the positioning error leads to clamping the electrode wire downward position, coupled with the electrode wire is very small bearing force, through the experiment the maximum force measured is only 0.3 N, which can easily cause damage to the electrode wire in the traction process, resulting in poor processing quality and high defect rates.

Figure 20a is a model of the second kind of micro-gripper designed. We hope to make a special shape at the top of the micro-gripper tip to encircle the electrode wire. Figure 20b is a picture of the machined micro-gripper, Figure 20c is a microscopic image of the tip structure of the micro-gripper, and Figure 20d is the state of the micro-gripper when clamping the electrode wire. In addition, although this micro-gripper was designed with the disadvantages of the micro-gripper in Figure 19 in mind, due to its rougher surface, the shape of the middle is no longer visible during the closing of the two grippers, which will produce extrusion damage to the electrode wire during the clamping of the wire, and in addition, traction manipulations in the state of the extruded electrode wire will result in damage to the electrode wire or pulling off the phenomenon of The traction of the wire in a squeezed state can lead to damage or breakage of the wire. It can be seen from Figure 20c that the surface of the micro-gripper tip is also relatively rough, and the distortion of the shape is relatively high. Compared with the silicon micro-gripper in Figure 15, the shape integrity is poor. Similar to the micro-gripper in Figure 19, when the width of the micro-gripper tip is small, the tip rigidity of the micro-gripper is poor, and it is easy to break.

By comparing the silicon micro-gripper and steel micro-gripper in Figure 15, Figure 19 and Figure 20, it can be seen that when the geometric dimensions such as the width and thickness of the tip are small (tens of microns to hundreds of microns), the silicon micro-gripper has more advantages in shape integrity and mechanical properties.

## 5. Conclusions

In this paper, a micro-gripper with an envelope structure is designed to capture, pull and release small diameter (tens of microns) flexible wires. The opening-closing displacement and speed of the microgripper are controlled by the screw drive. The values of these two indexes can be easily adjusted according to the manipulation requirements. Through the simulation and experiment of mechanical indexes, the following conclusions are obtained:

(1) The tip region adopts the enveloping-type design. The micro-gripper catches from the root of the electrode wire. After the tip-enveloping region is closed, the flexible electrode wire is pulled to the top of the pad and released by the movement of the micro-gripper. This manipulation mode is more stable than that of grasping the floating end;
(2) The experimental results show that the maximum bearing force of the flexible electrode wire with a diameter of 70 μm is about 0.3 N. Under this reaction force, the deformation of the tip-envelope region is about 27.52 μm, and the wire will not be lost in the process of pulling the electrode wire;
(3) Compared with the steel micro-gripper, the silicon micro-gripper with the same geometry has more advantages in shape integrity, machinability and mechanical properties. The deep silicon micromachining process can be used to fabricate the micro-gripper with a more complex structure and shape.

## Figures and Tables

**Table 1:** Geometric dimensions of coreless motor’s winding.

Parameter	Φ_1_ (mm)	Φ_2_ (mm)	H (mm)	L_1_ (mm)	Φ_3_ (mm)	Φ_4_ (mm)	Electrode Pad’s Size (mm)
Value	0.05~0.07	3	11~12	1.5~2	1	2.5	0.48

**Table 2:** Design parameters of micro-gripping system.

Material of Micro-Gripper	Clamping Mode	Opening-Closing Range (mm)	Computational Load (N)	Maximal Moving Distance (mm)	MOCV (mm/s)
Silicon	Envelope type	0~3	0.5	1	0.3

Where MOCV = Maximal opening-closing velocity.

**Table 3:** Comparison of Opening-closing displacement and speed results for different parameters.

n	400			800			1200			1200		
*f* (Hz)	100	200	300	100	200	300	60	120	180	360	720	1080
*v* (mm/s)	0.25	0.5	0.75	0.25	0.5	0.75	0.15	0.3	0.45	0.9	1.8	2.7
*L* (mm)	1	1	1	2	2	2	3	3	3	3	3	3

**Table 4:** Performance of iron, stainless steel and silicon.

Material	Density (g/cm^3^)	Tensile Strength (GPa)	Hardness	Yield Strength (MPa)
Iron	7.86	0.4	120 (Brinell hardness)	250
Stainless steel	7.93	0.52	187 (Brinell hardness)	205
Single-crystal silicon	2.33	17.19	7 (Mohs Hardness)	120

**Table 5:** Dimensions of microgripper.

Parameter	*L* (mm)	*W* (mm)	*H* (mm)	*W_t_* (mm)	*L_t_* (mm)	*C*_1_ and *C*_2_ (mm)
Value	25	13	0.1~0.4	0.14~0.21	0.14~0.28	0.07~0.14

**Table 6:** Results of bearing capacity measurement for electrode wires.

Interval for *θ* (°)	Sample Number	Maximum Bearing Force		
		*F*_w1_ (N)	*F*_w2_ (N)	*F*_w3_ (N)
**0–5°**	**S1**	0.242	0.246	0.254
	**S2**	0.254	0.29	0.146
**10–15°**	**S3**	0.229	0.234	0.236
	**S4**	0.229	0.275	0.208
**40–50°**	**S5**	0.229	0.134	0.147
	**S6**	0.175	0.195	0.201

Where the subscripts w1–w3 represent wire number.

**Table 7:** Results of deformation measurement via different pulling force.

Pulling Force (N)	0	0.1	0.2	0.3	0.4	0.5	0.6
**Deformation symbol**	*L*_0_	*L*_1_	*L*_2_	*L*_3_	*L*_4_	*L*_5_	*L*_6_
**Deformation in image (pixel)**	0	5	11	16	18	20	25
**Deformation in space (μm)**	0	8.6	18.9	27.5	31.0	34.4	43.0
